# Widespread Contribution of Gdf7 Lineage to Cerebellar Cell Types and Implications for Hedgehog-Driven Medulloblastoma Formation

**DOI:** 10.1371/journal.pone.0035541

**Published:** 2012-04-23

**Authors:** Frances Y. Cheng, Xi Huang, Anuraag Sarangi, Tatiana Ketova, Michael K. Cooper, Ying Litingtung, Chin Chiang

**Affiliations:** 1 Department of Cell and Developmental Biology, Vanderbilt University Medical Center, Nashville, Tennessee, United States of America; 2 Department of Neurology, Vanderbilt University Medical Center, Nashville, Tennessee, United States of America; 3 Neuroscience Program, Vanderbilt University, Nashville, Tennessee, United States of America; University of Michigan, United States of America

## Abstract

The roof plate is a specialized embryonic midline tissue of the central nervous system that functions as a signaling center regulating dorsal neural patterning. In the developing hindbrain, roof plate cells express Gdf7 and previous genetic fate mapping studies showed that these cells contribute mostly to non-neural choroid plexus epithelium. We demonstrate here that constitutive activation of the Sonic hedgehog signaling pathway in the Gdf7 lineage invariably leads to medulloblastoma. Lineage tracing analysis reveals that Gdf7-lineage cells not only are a source of choroid plexus epithelial cells, but are also present in the cerebellar rhombic lip and contribute to a subset of cerebellar granule neuron precursors, the presumed cell-of-origin for Sonic hedgehog-driven medulloblastoma. We further show that Gdf7-lineage cells also contribute to multiple neuronal and glial cell types in the cerebellum, including glutamatergic granule neurons, unipolar brush cells, Purkinje neurons, GABAergic interneurons, Bergmann glial cells, and white matter astrocytes. These findings establish hindbrain roof plate as a novel source of diverse neural cell types in the cerebellum that is also susceptible to oncogenic transformation by deregulated Sonic hedgehog signaling.

## Introduction

The roof plate is a transient embryonic dorsal midline epithelial tissue spanning the entire developing central nervous system (CNS). The LIM-homeodomain transcription factor Lmx1a is a central regulator of roof plate development, as loss of Lmx1a resulted in a major absence of roof plate cells during early embryogenesis [1], [2]. The roof plate consists of a distinct strip of the most dorsal-lateral neuroectodermal cells that collectively function as an essential organizing center regulating development of neighboring tissues. Roof plate-derived inductive signals, such as Bmp6, Bmp7, Gdf7 (Bmp12), and Wnt1 [1], [3], [4], are important for directing differentiation of dorsal neuronal cell types [1], [3], [4], [5]. While regulating the development of neighboring tissues by secreted growth factors, the roof plate cells also have the capacity to generate different cell types. For example, in the spinal cord region, Gdf7-expressing roof plate progenitors give rise to dorsal interneurons and neural crest-derived sensory neurons and glia [3], [6]. In the telencephalon, roof plate progenitor cells have been implicated as a source of marginal zone neurons [7]. Although the roof plate varies in its differentiation potential along the rostral-caudal axis of the neural tube [8], [9], [10], previous fate-mapping studies have indicated that the hindbrain roof plate is uniquely restricted in lineage potential and its contributions are mostly limited to non-neural hindbrain choroid plexus epithelial (hChPe) cells [5], [9], [10], [11], [12]. Hence, the capacity for hindbrain roof plate Gdf7-expressing cells to contribute to specific cell types in the cerebellum has not been shown.

Adjacent to the hindbrain roof plate is the cerebellar rhombic lip, which is a source of migratory neurons that primarily stream towards the cerebellar anlage to form multiple cell types. Rhombic lip derivatives include neurons of the deep cerebellar nuclei, granule neuron progenitors, and unipolar brush cells, each arising within a specific developmental time window. Previous genetic analyses of the cerebellar rhombic lip have suggested that the basic helix-loop-helix transcription factor Mouse atonal homolog 1 (Math1) molecularly defines the region of the rhombic lip [13], [14]. However, more recent investigation has shown the rhombic lip to be molecularly heterogeneous with Lmx1a expression representing at least one Math1-independent rhombic lip gene [12]. Thus, the extent and contribution of Math1-negative cell types residing in the rhombic lip has yet to be elucidated.

Medulloblastoma, the most common malignant brain tumor in children, is characterized by its rapid progression and tendency to spread along the entire brain-spinal axis with poor clinical outcome. Recent integrative transcriptional profiling studies have showed that medulloblastoma comprises a collection of four clinically and molecularly diverse subgroups [15], [16], [17], [18], [19], [20]. Two of these subgroups, molecularly defined by overactivated WNT or SHH signaling, consistently demonstrate distinct genetic profiles and recently were found to arise from different cellular origins [21]. It is now well established that Sonic hedgehog (Shh) signaling stimulates proliferation of cerebellar granule neuron precursors (CGNPs) during cerebellar development [22], [23], [24], [25]. Numerous studies using mouse models in which the Shh pathway is constitutively activated have linked Shh signaling to medulloblastoma and CGNPs as a cellular origin [26], [27], [28]. Notably, the majority of Shh-driven mouse models involve transformation of a large number of neural stem cells (GFAP) [27], neural progenitors (Nestin) [29], [30] or CGNPs (Math1) [28]. To date there have been few distinct subsets of CGNPs identified which can be transformed to initiate medulloblastoma formation, with the exception of Olig2- and Tlx3-expressing precursors [28]. Further identification of the cellular origins of medulloblastoma may help to better understand early developmental pathways involved in tumorigenesis and focus treatment on cell types responsible for tumor initiation.

In our previous study, we observed that Gdf7^Cre/+^;SmoM2 mutant mice, in addition to demonstrating enhanced proliferation of the hindbrain choroid plexus epithelial progenitor cells, are runted and exhibit neurological defects [31]. Here we show that ectopic Shh signaling in the *Gdf7*-lineage cells invariably led to formation of medulloblastoma with CGNP features, indicating that focal activation of the Shh signaling pathway in the Gdf7-lineage cells is sufficient to promote cerebellar tumorigenesis. This result is at odds with previous findings that hindbrain roof plate may only contribute to non-neural choroid plexus epithelium. Using lineage tracing analysis, we demonstrate that in addition to their contribution to the choroid plexus epithelium, *Gdf7*-expressing cells are a source of distinct progenitor populations in the rhombic lip and dorsal midline cerebellar ventricular zone. These populations contribute to multiple cerebellar neuronal and glial cell types, including CGNPs, the presumed cell of origin for hedgehog-driven medulloblastoma. Our findings uncover a broad contribution of Gdf7-lineage to the cerebellum and suggest that medulloblastoma can stem from progenitor populations that were previously thought to be restricted to the choroid plexus lineage.

## Results

### Targeted activation of Shh signaling pathway in Gdf7-lineage leads to rapid cerebellar hyperplasia

**Figure 1:**
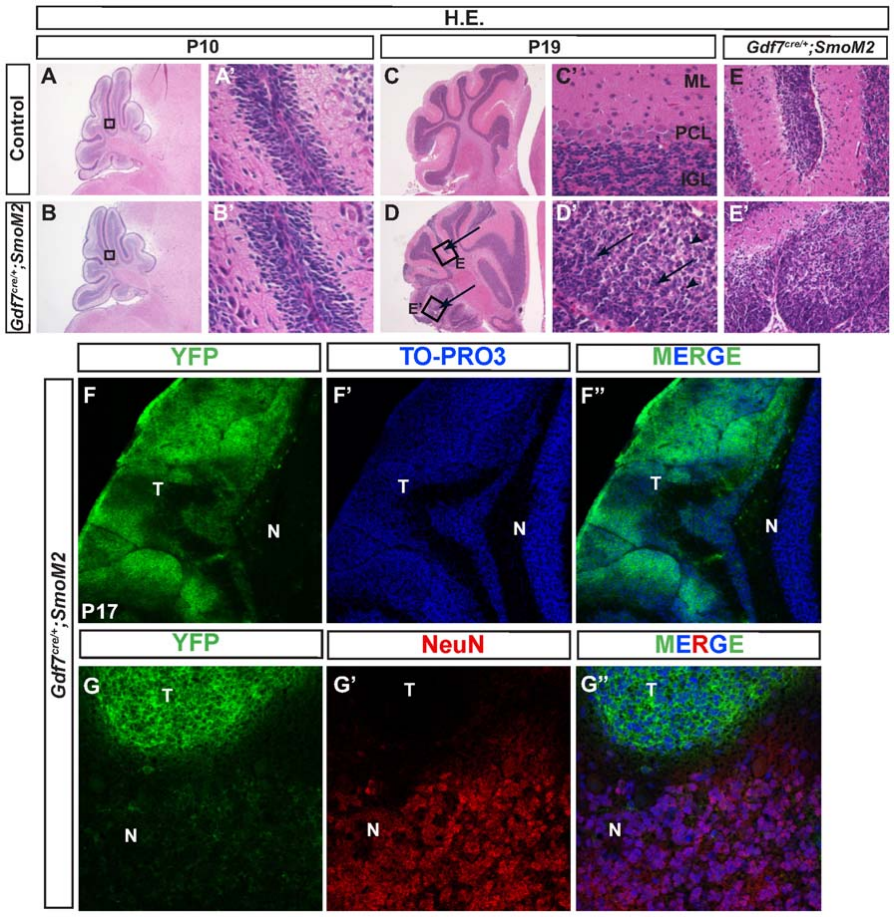
Shh pathway activation in Gdf7-lineage cells leads to cerebellar hyperplasia. *Gdf7^Cre/+^;SmoM2* gain-of-function mutant mice exhibit cerebellar defects. (A–B′) Hematoxylin-eosin staining of wild-type and *Gdf7^Cre/+^;SmoM2* mutants. Prior to P10 histological sections of mutants are similar to control. (C–E′) *Gdf7^Cre/+^;SmoM2* mutants over 14 days old develop ectopic foci of densely packed cells within the molecular layer of their cerebella. Higher magnification view of these foci reveals no discernible layer organization and resemblance to neoplastic lesions. Arrows in (D) indicate regions of hypercellularity. Boxed regions E and E″ are magnified and shown in the right adjacent panels. Arrows in D′ indicate nuclear molding. Arrowheads in D′ indicate apoptotic nuclei. (F-F″) The ectopic foci consist of cells of the Gdf7-lineage as indicated by their expression of SmoM2-YFP. (G-G″) Ectopic foci do not express differentiated neuronal marker NeuN. Abbreviations: EGL, external granular layer. ML, molecular layer. IGL, internal granular layer. T, tumor region. N, normal cerebellum.

We recently reported that the hChPe cells robustly express Shh and Shh signaling defines a discrete hChPe progenitor domain close to the lower rhombic lip [31]. To support a crucial proliferative role for Shh signaling during hChPe development, we generated *Gdf7^Cre/+^;SmoM2* mutants in which *Gdf7^Cre^* drives constitutively active Shh signaling in a ligand-independent manner due to a point mutation in the Smo allele [32], [33]. In line with the regulation of Shh signaling in the biogenesis of the hChP, we observed enlarged hChP in the gain-of-function *Gdf7^Cre/+^;SmoM2* mutant mice. Notably, *Gdf7^Cre/+^;SmoM2* mice displayed stunted growth, cranial bulging in the hindbrain region, and impaired motor coordination [31]. As reported in our previous study, all of the *Gdf7^Cre/+^;SmoM2* mice died within three weeks of birth with a median survival of 13.5 days [31]. Dissected *Gdf7^Cre/+^;SmoM2* cerebella often lacked visible foliations, suggesting that the spaces between cerebellar lobules were filled with cellular material. Examination of hindbrain histology in surviving pups prior to P10 revealed proper development of all cerebellar layers and relatively normal architecture (Figure 1A–B′). However, hematoxylin and eosin staining of tissue sections from mice surviving beyond P14 revealed tumors within the cerebellar parenchyma and leptomeninges (Figure 1C–E′, arrows). The cells were pleomorphic with a high nuclear-to-cytoplasmic ratio. Nuclear molding was a common feature (Figure 1D′, arrows), as was the presence of apoptotic cells (Figure 1D′, arrowheads). In the parenchymal lesions, areas of relatively preserved molecular layer architecture with hypercellularity were suggestive of persistence of the external granule layer (EGL) (Figure 1E-E′). As the membrane-localized SmoM2 protein is fused with a YFP reporter protein [32], [34], we determined that the medulloblastoma cells were all YFP-positive (Figure 1F-F″). Furthermore, YFP expression covering most of the cerebellar surface in the *Gdf7^Cre/+^;SmoM2* mice suggests that the tumor cells are derived from Gdf7-expressing progenitor cells. Importantly, YFP-positive cells do not express the differentiated neuronal marker NeuN (Figure 1G-G″). Collectively, these data indicate that targeting constitutively active Shh signaling to the Gdf7-lineage leads to the formation of medulloblastoma.

### *Gdf7^Cre/+^;SmoM2* medulloblastomas display cerebellar granule neuron precursor features and similar molecular phenotypes to medulloblastomas in *Patched^LacZ/+^* mice

**Figure 2:**
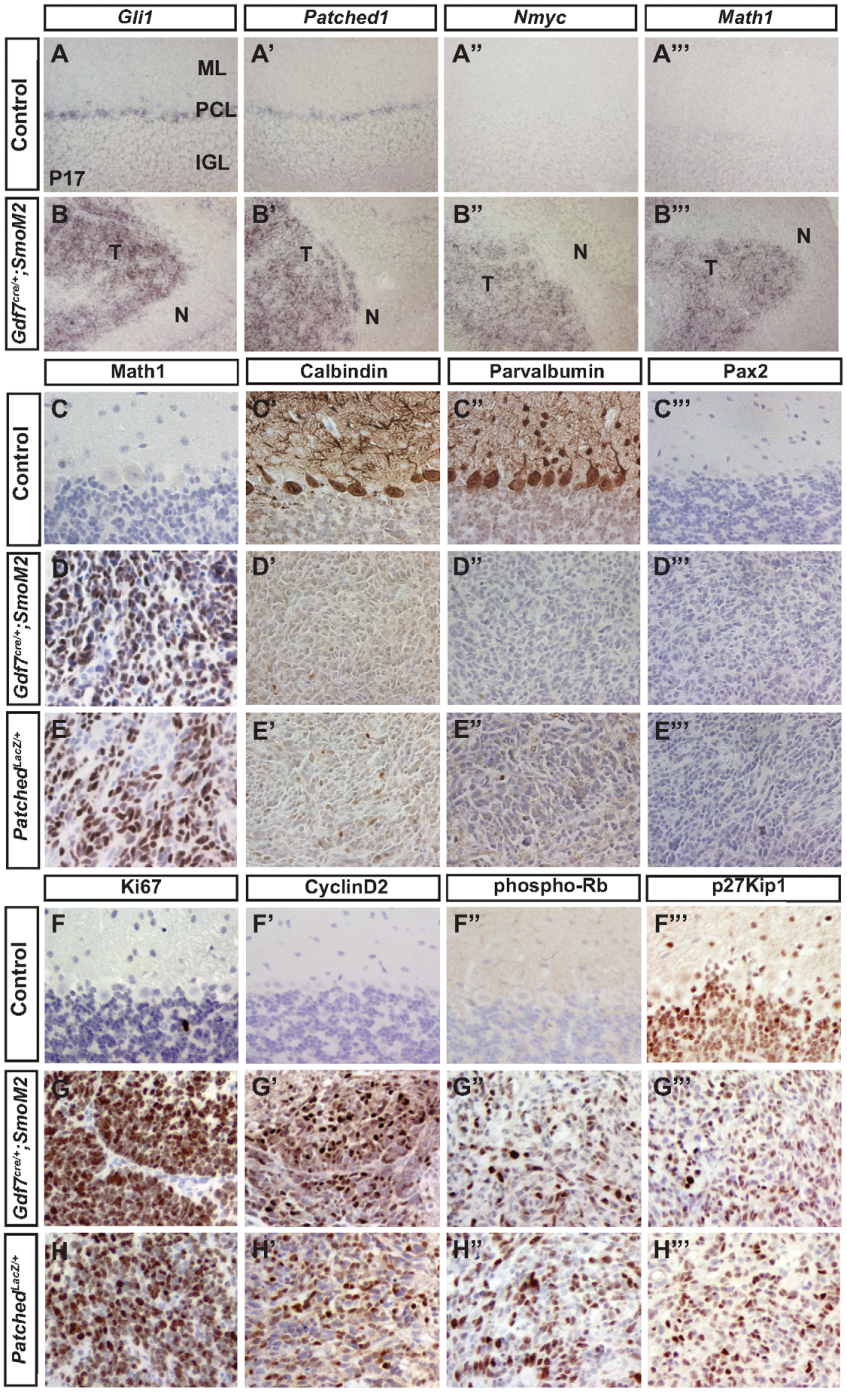
*Gdf7^Cre/^;SmoM2* mice develop medulloblastoma with CGNP features. (A–B″′) In situ hybridization of wild-type and *Gdf7^Cre/+^;SmoM2* mutants. The aberrant tissue foci of *Gdf7^Cre/+^;SmoM2* cerebella display high level Shh signaling as determined by *Gli1* and *Ptch1* expression, and *Nmyc* and *Math1* expression. (C–H″′) *Gdf7^Cre/+^;SmoM2* mice develop medulloblastoma consisting of cells of the CGNP fate. Tumors in *Gdf7^Cre/+^;SmoM2* mice and adult *Patched1^LacZ/+^;SmoM2* mice appear very similar. Abbreviations: ML, molecular layer. PCL, Purkinje cell layer. IGL, internal granular layer. T, tumor region. N, normal cerebellum.

Consistent with the fact that constitutively active SmoM2 was expressed in Gdf7-lineage cells and the tumor cells are marked by YFP, we detected a high level of Shh signaling, as evidenced by robust expression of pathway target genes *Gli1* and *Ptch1* in *Gdf7^Cre/+^;SmoM2* medulloblastomas (Figure 2B and 2B′). In contrast, moderate levels of *Gli1* and *Ptch1* were detected only in putative Bergmann glial cells of control cerebella (Figure 2A and A′). Emerging evidence suggests that Nmyc is an essential oncogenic mediator for Shh-dependent medulloblastoma [35], [36], [37], [38]. More importantly, a recent study demonstrated that Nmyc promotes progression from preneoplastic lesions to medulloblastoma [39]. While *Nmyc* expression was not detectable in the cerebella of control mice older than 2 weeks, robust expression was measured in *Gdf7^Cre/+^;SmoM2* medulloblastoma cells (Figure 2A″ and 2B″), consistent with oncogenic transformation. Previous studies have shown that acquisition of CGNP fate is a prerequisite for medullollastoma formation [27], [28]. As oncogenic transformation of CGNPs has been faithfully modeled in the *Patched^LacZ/+^* mice [40], [41], we compared medulloblastomas in these mice to those in the *Gdf7^Cre/+^;SmoM2* mice. Notably, tumors from both *Gdf7^Cre/+^;SmoM2* and *Patched^LacZ/+^* mice displayed strong expression of Math1, a marker of cells of the CGNP fate (Figure 2C, 2D, 2E), comparably low expression of calbindin-positive Purkinje neurons and parvalbumin-positive GABAergic interneurons, and absence of Pax2-positive GABAergic interneuron progenitors (Figure 2C-E″′). Similar to *Patched^LacZ/+^* tumors, *Gdf7^Cre/+^;SmoM2* medulloblastomas expressed the neural progenitor marker Nestin and were highly proliferative as indicated by strong expression of Ki67, CyclinD2, and phosphorylated Rb and partial loss of differentiation marker p27Kip1 (Figure 2F–H″′). Taken together, these data demonstrate that *Gdf7^Cre/+^;SmoM2* and *Patched^LacZ/+^* mice develop medulloblastomas with similar cellular and molecular phenotypes and CGNP identity.

### A subset of Gdf7-lineage tumor cells are multipotent

**Figure 3:**
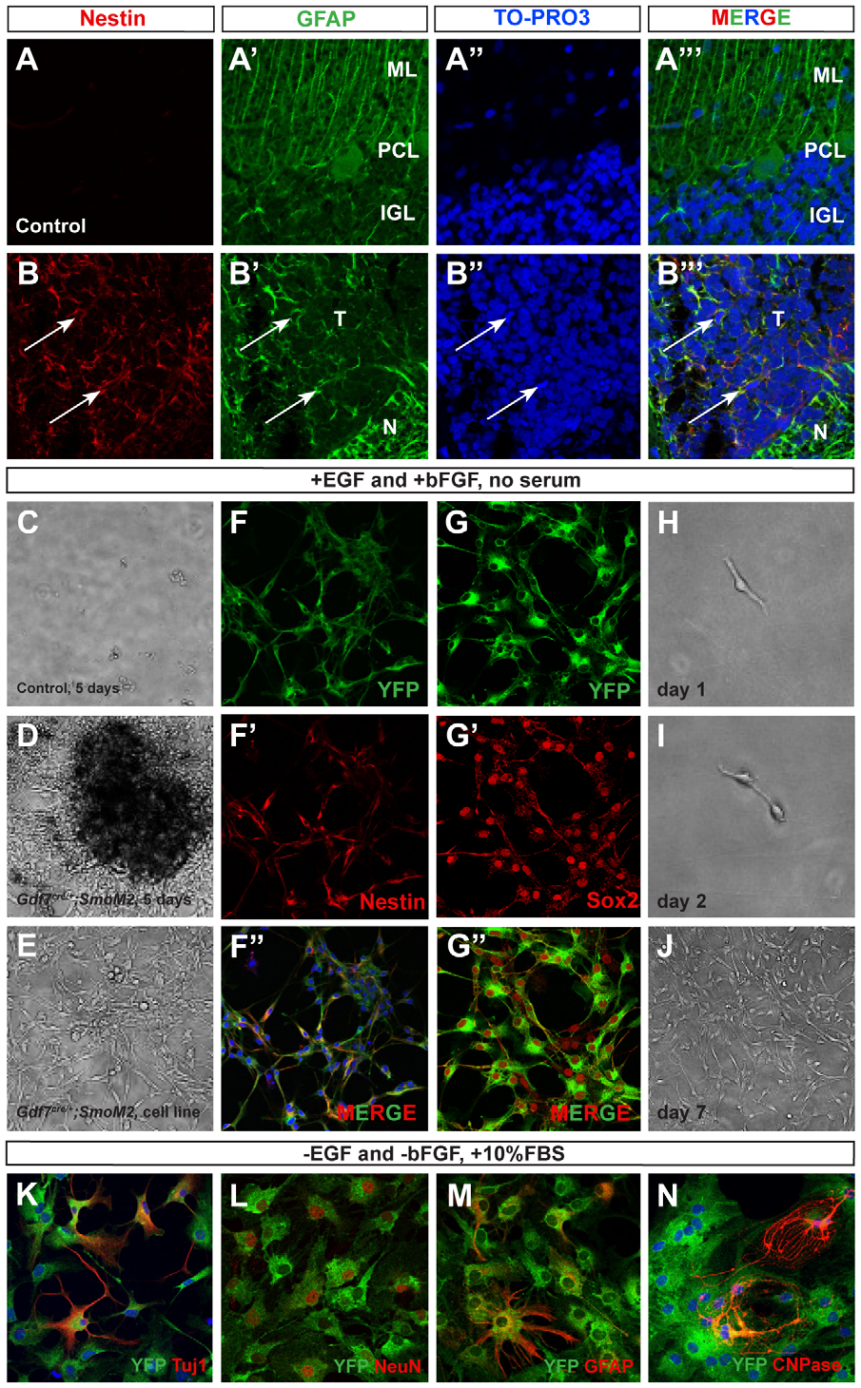
A subset of Gdf7-lineage cells express neural stem cell markers. (A–B″′) Many cells within the tumor tissue of *Gdf7^Cre/+^;SmoM2* mice coexpress neural stem cell marker Nestin (red) and glial marker GFAP (green). Arrows indicate co-localization. (C–E) Tumor cell lines can be invariably established from *Gdf7^Cre/+^;SmoM2* cerebella. (F–G″) These cells highly express multiple neural stem cell markers Nestin and Sox2 and can undergo serial passages. (H–J) Here, one representative colony is shown when cultured for 3 and 6 days. (K–N) *Gdf7^Cre/+^;SmoM2* cultured cells can differentiate into mature cerebellar cell types upon switching to culture media containing 10% FBS.

A recent report has shown that a subset of medulloblastoma cells from *Patched1^LacZ/+^* mice are multipotent progenitors and capable of differentiating into glial and neuronal lineages [42]. To determine whether the medulloblastoma cells in *Gdf7^Cre/+^;SmoM2* mice possessed similar properties, we assayed for the presence of potential cancer stem-cell like markers. Consistently, we were able to detect Nestin+ and GFAP+ cells in *Gdf7^Cre/+^;SmoM2* tumor foci, suggestive of a tumor stem cell immunophenotype (Figure 3A–B″′, arrows). We then dissected the cerebella from *Gdf7^Cre/+^;SmoM2* mice that older than 14 days and from age-matched control mice. Cerebellar cells were dissociated and cultured in neural stem cell medium. While we did not observe appreciable colony formation by the dissociated control cerebellar cells, we observed the formation of numerous highly proliferative colonies within days from every mutant cerebellum analyzed (Figure 3C–E). These cells expressed the neural stem cell markers Nestin, Sox2, and GFAP, sustained multiple serial passages (over 50) and were clonogenic at a plating density of 100 cells per ml culture medium (Figure 3F–J). Under stem cell culture conditions, the YFP+ cells from *Gdf7^Cre/+^;SmoM2* medulloblastomas were small and bipolar with large nuclei and scant cytoplasm (Figure 3I). Upon switching to medium supplemented with 10% fetal bovine serum, the YFP+ cells displayed dramatically altered morphology within 5–7 days, withdrew from the cell cycle, and differentiated into Tuj1+ or NeuN+ neurons, GFAP+ astrocytes or CNPase+ oligodendrocytes, highlighting their multi-potency (Figure 3K–N). These data suggest that a subset of Gdf7-lineage cells in the *Gdf7^Cre/+^;SmoM2* tumors express multiple stem cell markers and are clonogenic and multipotent, reported characteristics for the medulloblastoma-propagating cells of the *Patched^LacZ/+^* mouse model [42].

### Distinct Gdf7-expressing cells of the cerebellar vermal ventricular zone are radial glial cells

**Figure 4:**
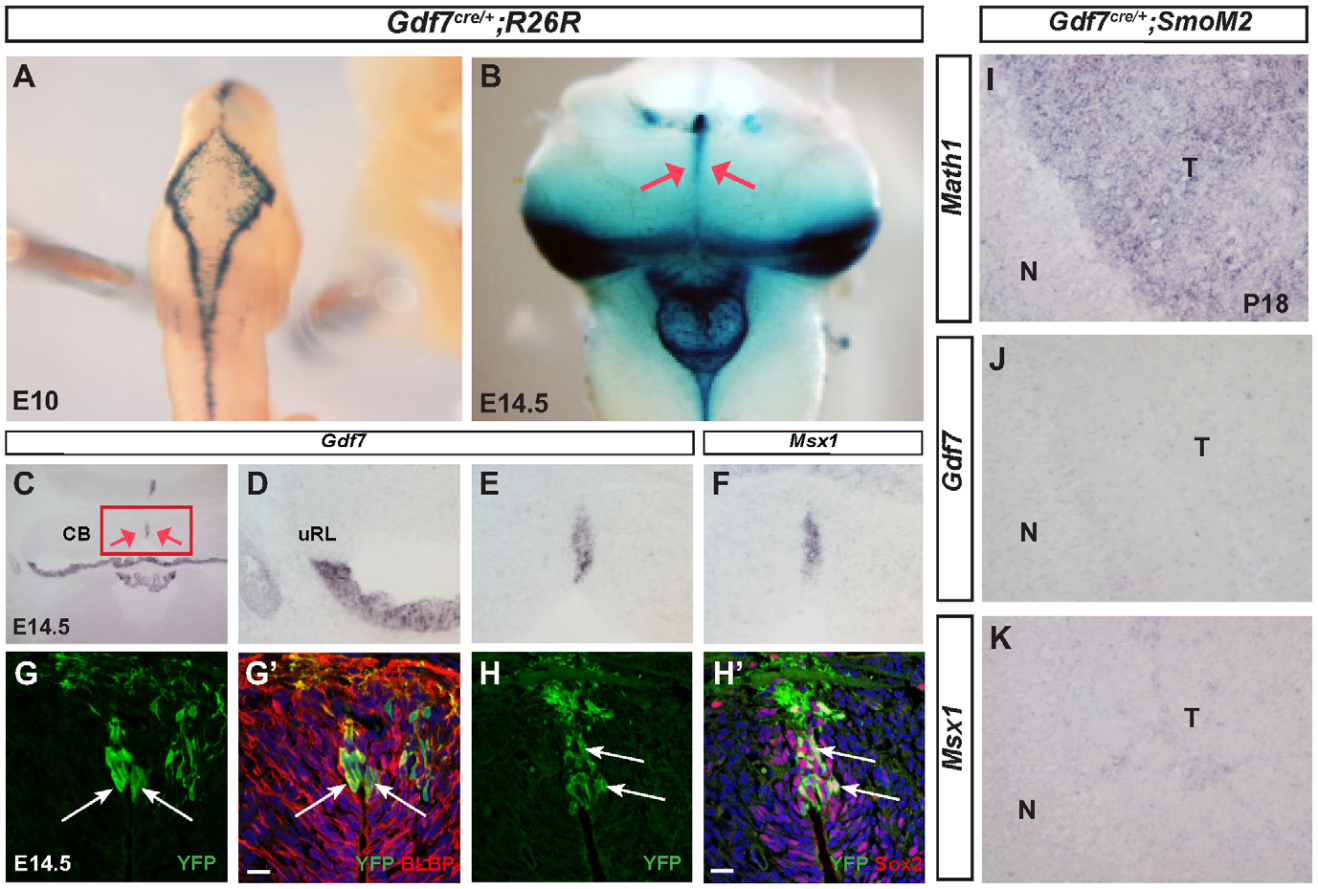
Roof plate cells give rise to a distinct population of cerebellar vermal radial glia cells. (A) Whole mount X-gal staining for ß-galactosidase in *Gdf7^Cre/+^;ROSA^lacZ^* embryos shows that Gdf7 lineage is present in the hindbrain roof plate at E10. (B) At E14.5, in addition to hindbrain choroid plexus, Gdf7 lineage also contributes to the ventricular zone of cerebellar vermis (red arrows). (C–F) In situ hybridization at E14.5 showing *Gdf7* and its transcriptional target *Msx1* are expressed in the cerebellar vermis. (G–H′) Fate-mapping of the Gdf7-lineage in *Gdf7^Cre/+^;ROSA^eYFP^* cerebella indicates that Gdf7 is present at E14.5 in a distinct population of vermal radial glia cells as highlighted by coexpression of YFP and BLBP (G-G′) or Sox2 (H-H′) Arrows indicate colocalization. (I–K) In situ hybridization at P18 shows that the *Math1+ Gdf7^Cre/+^;SmoM2* tumor tissue does not express *Gdf7* or *Msx1*. Abbreviations: N, normal cerebellar tissue. T, tumor tissue. CB, cerebellum. uRL, upper rhombic lip.

The unexpected finding that *Gdf7^Cre/+^;SmoM2* mice displayed cerebellar oncogenic transformation prompted us to ask whether the Gdf7-lineage cells only differentiate into mature hChPe cells as previously recognized, or contribute to other cerebellar cell types including CGNPs that are susceptible to oncogenic transformation by aberrant Shh signaling. Therefore, we performed detailed fate-mapping of the Gdf7 lineage in *Gdf7^Cre/+^;ROSA26^LacZ^* and *Gdf7^Cre/+^;ROSA26^eYFP^* mice, where LacZ or enhanced YFP indelibly marks cells that are, or once were, expressing Gdf7. As expected, Gdf7-lineage cells were distributed at the dorsal midline along the entire cranio-spinal axis (Figure 4A, 4B). In this hindbrain region, Gdf7-lineage cells emanated from the lateral edge to occupy the medial portion of the roof plate, a migratory pattern similar to the reported *Ttr*-expressing primitive hChPe cells (Figure 4B) [43]. We observed a streak of Gdf7-lineage cells in the midline vermal cerebellar tissue where the two hemispheres meet (Figure 4C). This observation is consistent with the fact that we detected restricted *Gdf7*-expressing cells in the cerebellar midline tissue (Figure 4E), which persisted into E16.5 embryos (Figure S1). Interestingly, *Msx1*, a Bmp signaling target gene, was highly expressed in this cerebellar midline domain and suggestive of local signaling (Figure 4F). Furthermore, we observed that at embryonic day 14.5 (E14.5), all Gdf7-lineage cells localized to the vermal ventricular zone expressed radial glial cell markers BLBP and Sox2 (Figure 4G–H′). Radial glial cells have been shown to be multipotent neural stem cells during embryogenesis [44]. These data suggested that the Gdf7-expressing cerebellar vermal cells are a distinct sub-population of multipotent radial glial cells. As radial glial cells are rapidly proliferating during embryonic stages, we asked whether Gdf7 could act as a proliferative signal. The lack of apparent Gdf7 and Msx1 expression in the Math1+ tumor tissue of *Gdf7^Cre/+^;SmoM2* mice argues against this possibility (Figure 4I–K).

### Gdf7-lineage cells of the cerebellar rhombic lip are Lmx1a+ neural progenitor cells that likely give rise to CGNPs

**Figure 5:**
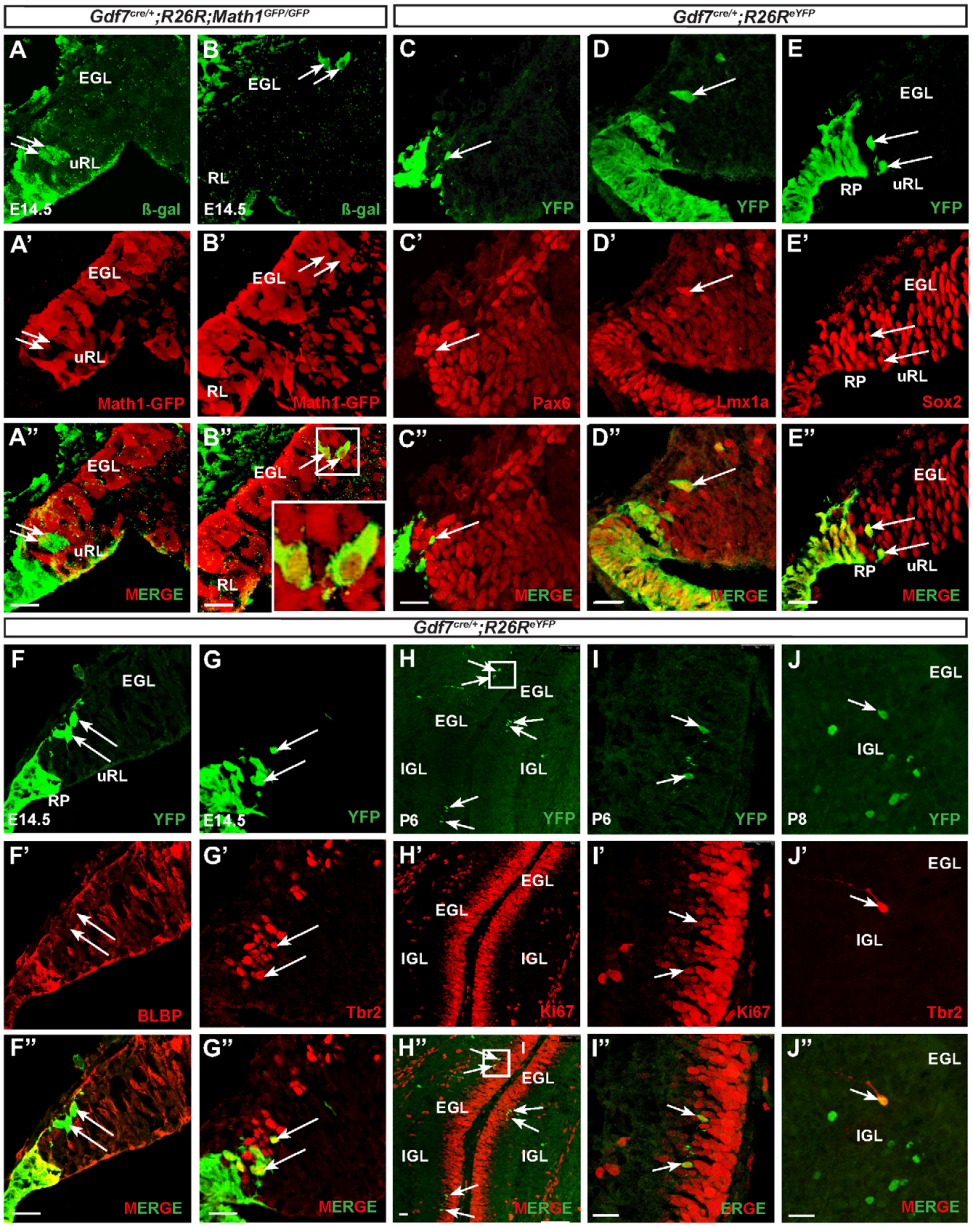
Gdf7-lineage cells in the cerebellar rhombic lip are Lmx1a+ neural progenitor cells that give rise to CGNPs. (A–B″) Fate-mapping studies in *Gdf7^Cre/+^;ROSA26^eYFP^;Math1^GFP/GFP^* at E14.5 show that Gdf7-lineage cells (as indicated by b-gal, green) are Math1-negative (as indicated by GFP) in sagittal sections of the upper rhombic lip. However these cells retain the capacity to express Math1 in the EGL. Arrows indicate delaminating Gdf7-lineage cells from the upper rhombic lip en route to the EGL. (C–F″) Fate-mapping studies in *Gdf7^Cre/^;ROSA26^eYFP^* mice at E14.5 show that delaminating Gdf7-lineage cells (green, arrows) are present in sagittal sections of the cerebellar rhombic lip and are Pax6- (C″, red), Lmx1a+ (D″, red), Sox2+ (E″, red), and BLBP- (F″, red). (G-G″) Some delaminating cells are also Tbr2+. Arrows indicate delaminating YFP-positive cells. (H–I″) Fate-mapping studies in P6 sagittal sections show co-localization of YFP and Ki67 (red), demonstrating that Gdf7-lineage cells contribute to proliferating cells in the EGL. Arrows indicate double-positive cells. Rectangle in (H) indicates enlarged area in (I). (J-J″) Tbr2-immunostaining at P8 demonstrates that Gdf7-lineage cells can also contribute to the cerebellar population of unipolar brush cells. Abbreviations: EGL, external granular layer. uRL, upper rhombic lip. RP, roof plate. Scale bar, 20 µm.

As expected, at stage E14.5 a second population of Gdf7-lineage cells was present in the laterally positioned roof plates adjacent to the choroid plexus. Surprisingly, several Gdf7-lineage cells delaminated from the clustered roof plate cells to enter the rhombic lip (Figure 5A). The number of delaminating Gdf7-lineage cells was few but distinct, amounting to no more than 20–30 cells per embryo at E14.5. In order to determine the identity of these cells, we sought to co-localize them with Math1, a molecular marker of the rhombic lip [13]. Using *Gdf7^Cre/+^;ROSA26^LacZ^*;*Math1^GFP/GFP^* in which GFP marks cells expressing Math1 and β-gal labels cells that once were or are expressing Gdf7, we found that the Gdf7-lineage cells in the rhombic lip were Math1-negative, corroborating recent evidence that molecular heterogeneity exists within the cerebellar RL (Figure 5A-A″) [12]. Accordingly, the cells were negative for Pax6, a marker for CGNPs (Figure 5C-C″). However, Gdf7-lineage cells retain the capacity to express Math1 as they migrate into the EGL (Figure 5B-B″). Because Lmx1a is currently the only known Math1-independent rhombic lip gene [12], we sought to colocalize the Gdf7-expressing progeny with Lmx1a and found that they were indeed Lmx1a-positive (Figure 5D-D″). To further characterize the identity of the delaminated Gdf7-lineage cells in the rhombic lip, we determined their expression of Sox2, a marker for neural progenitor cells (Figure 5E-E″) and also found that they are BLBP-negative (Figure 5F-F″). Some GFP-positive cells co-labeled with Tbr2 (Figure 5G-G″, arrows), a marker for unipolar brush cells (UBC) which also originate from the rhombic lip [45], suggesting that Gdf7-lineage cells can also contribute to the UBC population. We then analyzed the fate of Gdf7-lineage cells at postnatal stages. Consistent with the Math1+ YFP cells at E14.5, we detected apparent YFP signal in the EGL and other tissue layers of the P6 cerebellum (Figure 5H, 5I, data not shown). The YFP positive cells in the EGL were Ki67 positive, indicating that they are cycling, proliferative CGNPs (Figure 5H–I″). These findings strongly suggest that progeny of Gdf7-expressing cells can enter the rhombic lip and eventually the EGL to become Math1+ CGNPs. Additionally, we observed YFP positive cells in the IGL that were also Tbr2 positive, demonstrating that Gdf7 lineage cells can become mature UBCs (Figure 5J-J″).

### Gdf7-lineage cells contribute to an extensive array of mature cerebellar cell types

**Figure 6:**
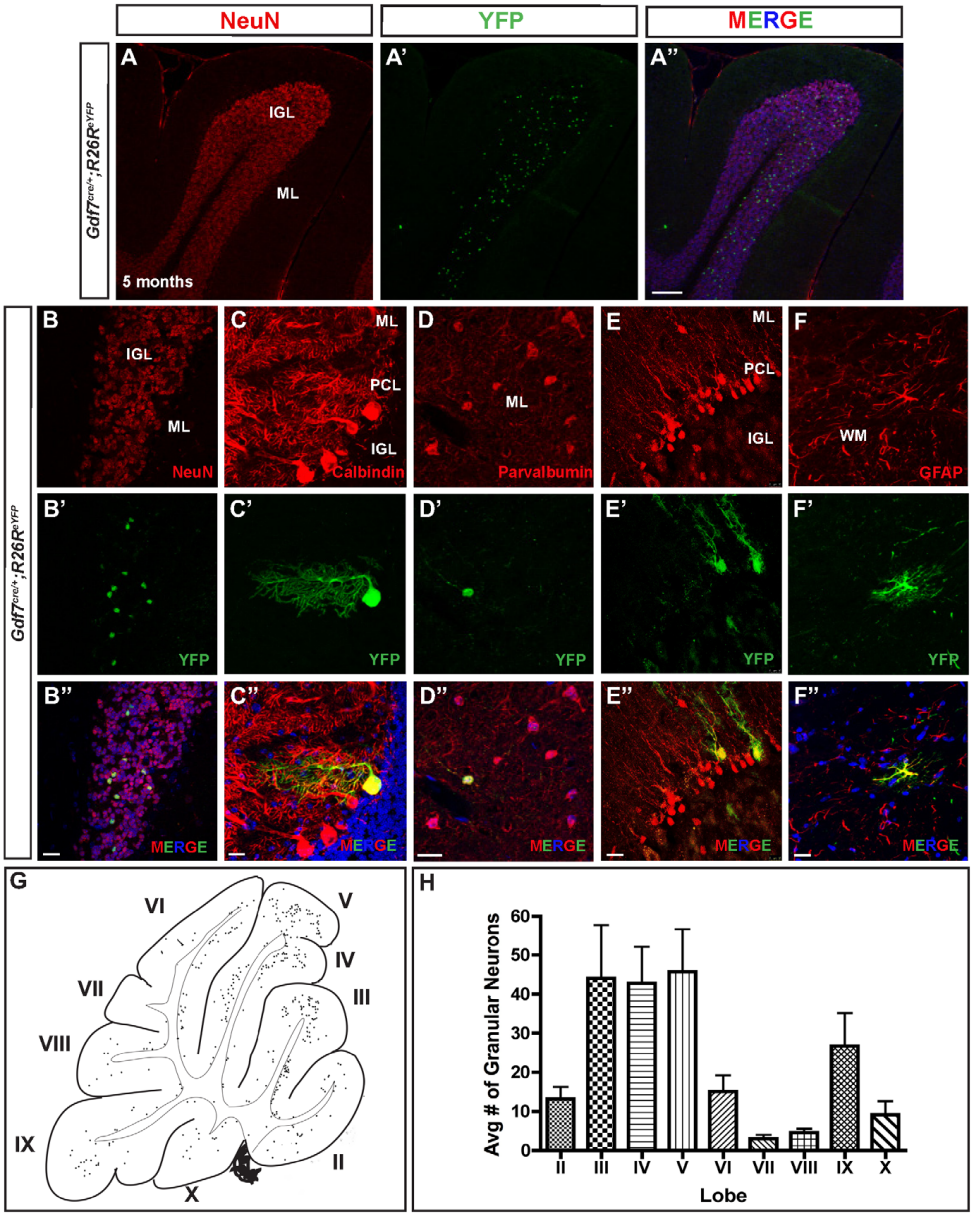
Gdf7-lineage cells contribute to an extensive array of mature cerebellar cell types. (A–F″) Fate-mapping studies in *Gdf7^Cre/+^;ROSA26^eYFP^* adult mice (age 5 months) show that Gdf7-lineage cells distribute to all the mature cerebellar tissue layers and contribute to granule neurons (A–B″), Purkinje neurons (C-C″), GABAergic interneurons (D-D″), Bergmann glia (E-E″), white matter astrocytes (F-F″), as well as non-neural hindbrain choroid plexus epithelial cells (data not shown). (G) Representative image of distribution of Gdf7-lineage cells in the adult mouse cerebellum (age 5 months). The locations of Gdf7-lineage cell nuclei (dots) were mapped by tracing from digital images of sagittal sections. Thick lines indicate the cerebellar surface; thin lines indicate the boundary between cerebellar cortex and white matter. Gdf7-lineage cells were located mainly in the internal granular layer with fewer in the PCL and ML. Cells in the IGL were concentrated in lobules III–V and IX. Roman numerals indicate lobules of the vermis. (H) Quantification of YFP+ granule neurons in the IGL per lobule. Data are mean of *n* = 10 sagittal sections of *Gdf7^Cre/+^;ROSA26^eYFP^* age 5 months. Abbreviations: EGL, external granular layer. ML, molecular layer. IGL, internal granular layer. WM, white matter. Scale bar, 100 µm.

In line with their widespread distribution in P6 cerebellum, we found that the Gdf7-lineage cells contribute extensively to various mature cerebellar cell types. In *Gdf7^Cre/+^;ROSA26^eYFP^* cerebella from adult mice, YFP-marked cells co-expressed granule neuron marker NeuN (∼3.5%), Purkinje neuron marker calbindin (∼0.1%), GABAergic interneuron marker parvalbumin (<0.1%), Bergmann glia marker Sox2 (∼0.2%), and white matter astrocyte cell marker GFAP (<0.1%) (Figure 6A–F″). It is interesting to note that granule neurons were the predominant cellular derivatives. A similar situation has been reported indicating that Gdf7-expressing roof plate cells of the spinal cord region preferentially become sensory neurons, a process suggested to be mediated by the function of Gdf7 itself [6]. Granule neurons derived from the Gdf7-lineage were not uniformly distributed among the cerebellar lobes, with higher numbers found in Lobes III–V and IX (Figure 6G, 6H).

## Discussion

In this study we have discovered that Gdf7 lineage cells contribute to diverse cell types in the cerebellum. This observation is surprising given that previous studies using the same *Gdf7^Cre/+^* driver line reported that Gdf7 lineage cells do not contribute to the cerebellum [3], [12]. The contribution of Gdf7 lineage cells constitutes a minor percentage of each cerebellar cell type (<0.1% to ∼3.5%), which likely explains the discrepancy in findings. Indeed, the cerebellar oncogenic transformation that we observed in *Gdf7^Cre/+^;SmoM2* mice served as a sensitive method for uncovering the important and broader lineage potential of hindbrain roof plate cells. Our studies indicate that the cerebellar cell types derived from Gdf7 lineage include both neuronal and non-neuronal cells that originate from two distinct Gdf7-expressing domains within rhombomere 1 (rh1). The first domain is located in the midline of the cerebellar ventricular zone and consists of a small cluster of cells expressing radial glial markers. As radial glial cells are multipotent progenitors that contribute to different cell types in the cerebellum [44], [46], and the cerebellar ventricular zone is the sole source of Purkinje cells and other GABAergic cell types including interneurons [24], these midline cells are likely the progenitors for Purkinje cells, GABAergic interneurons, Bergman glia, and astrocytes that we observed in our Gdf7-lineage analysis. Our data provides evidence that, similar to the cortex, midbrain, and spinal cord [3], [7], [47], the hindbrain roof plate has a conserved role in the generation of neurons and other cell types. This region can be molecularly defined by the expression of *Gdf7* and *Msx1* (Figure 4). We propose that this cerebellar vermal region is a previously undescribed source of neurons that is continuous with and extends beyond the cerebellar ventricular zone.

Gdf7, along with other Bmps, has been shown to be capable of inducing CGNP marker expression in cultured neural tissue [48]. In addition, genetic ablation of Gdf7-expressing cells using diptheria toxin results in complete loss of Math1+ cells and reduced numbers of Ptf1a, Lhx1/5, and Lmx1a-expressing cells and their improper positioning in the developing cerebellar anlage [5]. The fact that both *Gdf7* and *Msx1*, a readout of the Bmp signaling pathway, are expressed in this central vermal region (Figure 4) suggests that continuous local signaling is occurring. Thus there is a strong possibility that Gdf7 is involved in providing local and perhaps even autocrine signals for specification of cerebellar cellular subtypes originating from this vermal ventricular zone, in a manner consistent with its role in the differentiation of dorsal interneurons in the spinal cord [4] and in regulating the number of cortical ventricular zone progenitors in the developing telencephalon [7]. Indeed, *Gdf7* is present in the hindbrain dorsal midline beginning at E10.5 [5] until at least E16.5 (Figure 4, Figure S1); *Msx1* expression is present at least until E14.5 (Figure 4). Purkinje precursor cells continue to proliferate in the cerebellar ventricular zone from E10 to E13 [24], [49], after which they begin radial migration to form a monolayer in the adult cerebellum.

The other domain of Gdf7 expression occurs distal to the upper rhombic lip, in the hindbrain roof plate epithelial (hRPe) cells and incipient choroid plexus [5], [10] (Figure 4). Recent fate mapping studies showed that Lmx1a activity is required to prevent Gdf7-expressing hRPe cells from acquiring upper rhombic lip-derived neuronal lineages including CGNPs [12]. However, Lmx1a progenitors themselves also contribute to the neuronal lineage in the cerebellum [12]. Moreover, in contrast to posterior rhombomere-derived hRPe cells that express choroid plexus markers upon their emergence from the Gdf7+ lineage at E9.5, the rh1-derived hRPe cells remain molecularly naïve and do not begin to differentiate into hChPe until E13 [10]. This observation suggests that Gdf7+ progenitors distal to the upper rhombic lip are not necessarily restricted to the choroid plexus lineage. Indeed, we found delaminating Gdf7-lineage cells beyond the clustered roof plate cells in the rhombic lip as well as in the EGL. These lineage tracing studies provide evidence that hRPe cells normally give rise to CGNPs.

Our studies show that delaminating Gdf7-lineage cells in the rhombic lip are Math1−/Lmx1a+/Sox2+ neural progenitor cells. These findings support previous studies suggesting molecular heterogeneity within rhombic lip progenitors [12] rather than the classical definition of the rhombic lip as a homogenous Math1+ progenitor population [13], [14]. These cells however retain the capacity to express Math1 and eventually contribute to the Math1 lineage (Figure 5). A previous inducible genetic fate mapping study showed that RL progenitors born early (E13.5) give rise to granule cells that will populate the anterior lobes, whereas later born RL progenitors (E15.5 to E18.5) give rise to granule cells that will populate the posterior lobes [13]. Interestingly, we found Gdf7-lineage contribution to granule cells in all lobes with the majority being in the anterior lobes III–V and posterior lobe IX, suggesting that Gdf7-lineage CGNPs are generated unevenly throughout the duration of rhombic lip CGNP production. Another low-represented precursor type similar to Gdf7-lineage CGNPs is Olig2 precursor cells [28]. Olig2 lineage cells populate lateral caudal folia (lobes IX and X) and *Olig2-Cre;SmoM2* tumors correspondingly have a posterior lateral location. The factors responsible for induction of CGNP fate from hRPe progenitors requires further study.

CGNP cells originate from the upper rhombic lip and migrate tangentially to transiently occupy the EGL, and are the cell-of-origin for Shh-dependent medulloblastoma [27], [28]. The Shh-dependent mouse medulloblastomas harbor molecular signatures associated with CGNPs. While several hedgehog-driven medulloblastoma mouse models have been generated and use Nestin, GFAP, or Math1 as driver lines for constitutive Shh pathway activity [27], [28], [29], [30], our *Gdf7^Cre/+^;SmoM2* medulloblastoma mouse model demonstrates how remarkably few cells are sufficient for oncogenic transformation and tumor formation. Gdf7-lineage cells contribute to approximately 3.5% of granule neurons in the mature cerebellum; the number of delaminating Gdf7-lineage neural progenitor cells in the rhombic lip is far fewer, amounting to no more than 20–30 cells per embryo at E14.5. Furthermore, our study shows that Gdf7+ lineage cells originating from hRPe are also susceptible to oncogenic transformation in response to deregulated Shh pathway activation. Of note, we emphasize that the transformed cells are Gdf7+ lineage cells and not Gdf7+ expressing cells. Our mouse studies demonstrate that Gdf7 is not expressed in Shh MBs (Figure 4J); it is therefore unlikely that human SHH-MBs would express GDF7. Indeed, this observation was corroborated by an online database search that revealed no Gdf7 upregulation in human MBs across all molecular subgroups [16], [17], [18], [20], [50], [51] though this does not exclude the possibility that Gdf7 is expressed in a rare subset of MBs.

The multi-lineage nature of hRPe progenitors suggests that medulloblastoma originating from hRPe can be associated with aberrant choroid plexus function. Previously we have shown that Shh signaling is essential to promote hChPe expansion and that constitutive Shh pathway activation in the Gdf7 lineage can lead to an expanded choroid plexus [31]. It is estimated that about 15% children diagnosed with medulloblastoma also suffer from hydrocephalus [52]. While tumor obstruction of the fourth ventricle may account for some of the cases, a significant portion of patients continue to suffer from hydrocephalus after tumor resection [53]. Interestingly we found that overactivation of Shh pathway in the choroid plexus, while leading to an expanded proliferative domain, did not result in choroid plexus tumor formation (this study and [31]). However other signaling pathways, most notably the Notch pathway, have been implicated in the formation of choroid plexus neoplasias, as *Gdf7-Cre* driven expression of the activated ligand Notch_ICD_ [10] led to persistent proliferation of hChPe cells and retrovirus-driven expression of the ligand Notch3 [54] resulted in choroid plexus papillomas. As choroid plexus dysfunction is often linked to hydrocephalus [55], [56], it is tempting to speculate that a subset of medulloblastoma patients may have defective choroid plexus function [57] and thus, a common pathological pathway and cellular origin of these diseases.

## Methods

### Ethics Statement

All animal experiments were carried in accordance to protocols (M09-222 and M09-160) approved by the Vanderbilt University Animal Care and Use committee.

### Mouse strains

The generation of *Gdf7^Cre/+^* mice was described previously [58]. *SmoM2* [34], *ROSA26^LacZ^* [59], and *ROSA26^eYFP^* [60] mice were obtained from the Jackson Laboratory. *Gdf7^Cre/+^;SmoM2* mice were identified by their smaller size, bulging cranium and confirmed by genotyping. *Math1^GFP/GFP^* mice were obtained from Jane Johnson, University of Texas-Southwestern. Fate-mapping studies were performed on *Gdf7^Cre/+^; ROSA26^LacZ^* and *Gdf7^Cre/+^; ROSA26^eYFP^* mice. At least three animals from control and mutants were used for each morphological/molecular analysis shown in each figure.

### Histological analyses, immunohistochemistry and immunocytochemistry

Standard hematoxylin and eosin stainings were performed to compare the histological features of control and mutant mice. All immunohistochemical analyses were performed on sections collected from OCT- or paraffin-embedded tissues. Twenty minutes of antigen retrieval at 95C° with citrate buffer (pH 6.0) were included for all stainings on paraffin sections. The primary antibodies used were rabbit anti-GFP, (Molecular Probe, 1∶500), chicken anti-GFP, (Aves Labs, 1∶200), rabbit anti-Sox2, (Millipore, 1∶400), rabbit anti-BLBP, (Millipore, 1∶1000), rabbit anti-Pax6, (Covance, 1∶500), mouse anti-NeuN, (Millipore, 1∶200), mouse anti-Nestin, (Developmental Studies Hybridoma Bank, DSHB, 1∶50), mouse anti-Calbindin, (Swant, 1∶500), mouse anti-Parvalbumin, (Sigma, 1∶200), rabbit anti-Math1 (gift of Jane Johnson, 1∶400), rabbit anti-Ki67 (NeoMarkers, 1∶400), rabbit anti-GFAP (Neuromics, 1∶500), mouse anti-GFAP (Neuromics, 1∶200), mouse anti-Cyclin D2 (NeoMarkers, 1∶500), rabbit-anti-phospho-Rb (Ser807/811) (Cell Signaling, 1∶300), mouse-anti-p27Kip1 (Transduction Laboratories, 1∶1000), chicken anti-Tbr2 (Millipore, 1∶100), and rabbit anti-Lmx1a (gift of Michael German, University of California-San Francisco, 1∶200). Immunocytochemical stainings were performed on primary *Gdf7^Cre/+^;SmoM2* tumor cells (see below under Medulloblastoma cell culture) grown for 48 hours on gelatinized glass coverslips. The primary antibodies were rabbit anti-GFP, (Molecular Probes, 1∶2000), mouse anti-Nestin, (DSHB, 1∶500), mouse anti-Sox2, (Millipore, 1∶500) mouse anti-Tuj1, (Sigma, 1∶1000), mouse anti-NeuN, (Millipore, 1∶1000), mouse anti-GFAP, (Neuromics, 1∶1000) and mouse anti-CNPase, (Sigma, 1∶1000). All fluorescent images were taken using Zeiss LSM 510 confocal microscope. Independent stainings were performed on at least three animals for each marker and representative images are shown.

### X-gal staining and transcript detection

X-gal staining for ß-galactosidase was performed according to standard protocol. Section *in situ* hybridizations were performed on 20 micron frozen sections as previously described [61]. The following cDNAs were used as templates for synthesizing digoxygenin-labeled riboprobes: *Gdf7* (Tom Jessell, Columbia University), *Gli1* (C-C Hui, University of Toronto), *Patched1* (Matthew Scott, Stanford University), *Nmyc* (Mary E. Palko, NCI), *Math1* (ATCC, I.M.A.G.E. No. 6530849).

### Medulloblastoma cell culture

Medulloblastoma tissue dissociation and tumor cell culturing were performed essentially as previously described [62]. Specifically, tumor-bearing cerebella in *Gdf7^Cre/+^;SmoM2* mice over 14 days old were dissected in sterile, ice-cold PBS, minced with 50% Accutase in PBS for 5 minutes followed by repetitive pipeting with Pipetman (P1000) for 3 minutes, then cell pellets were collected after brief centrifugation. Pelleted cells were then resuspended in neural stem cell culture medium and plated in a gelatinized 60 mm tissue culture dish. The stem cell culture medium is composed of Neurobasal medium with glutamine, N2, B27, 25 ng/ml human EGF and 25 ng/ml basic FGF. Unattached cells were removed by changing medium on the following day after initial seeding, then medium was changed every four days. All experiments using primary mouse MB cells were performed within 3 passages. For differentiation conditions, EGF and bFGF were withdrawn and 10% FBS added for 5–7 days.

## Supporting Information

Figure S1 **(A–B) In situ hybridization at E16.5 shows** ***Gdf7*** **expression persists in the cerebellar vermis.**
(TIFF)
