# Supplementary figures and images for: Widespread Contribution of Gdf7 Lineage to Cerebellar Cell Types and Implications for Hedgehog-Driven Medulloblastoma Formation
Source: PLoS One. 2012 Apr 23;7(4):e35541. doi: 10.1371/journal.pone.0035541 (PMC3335071; doi:10.1371/journal.pone.0035541)

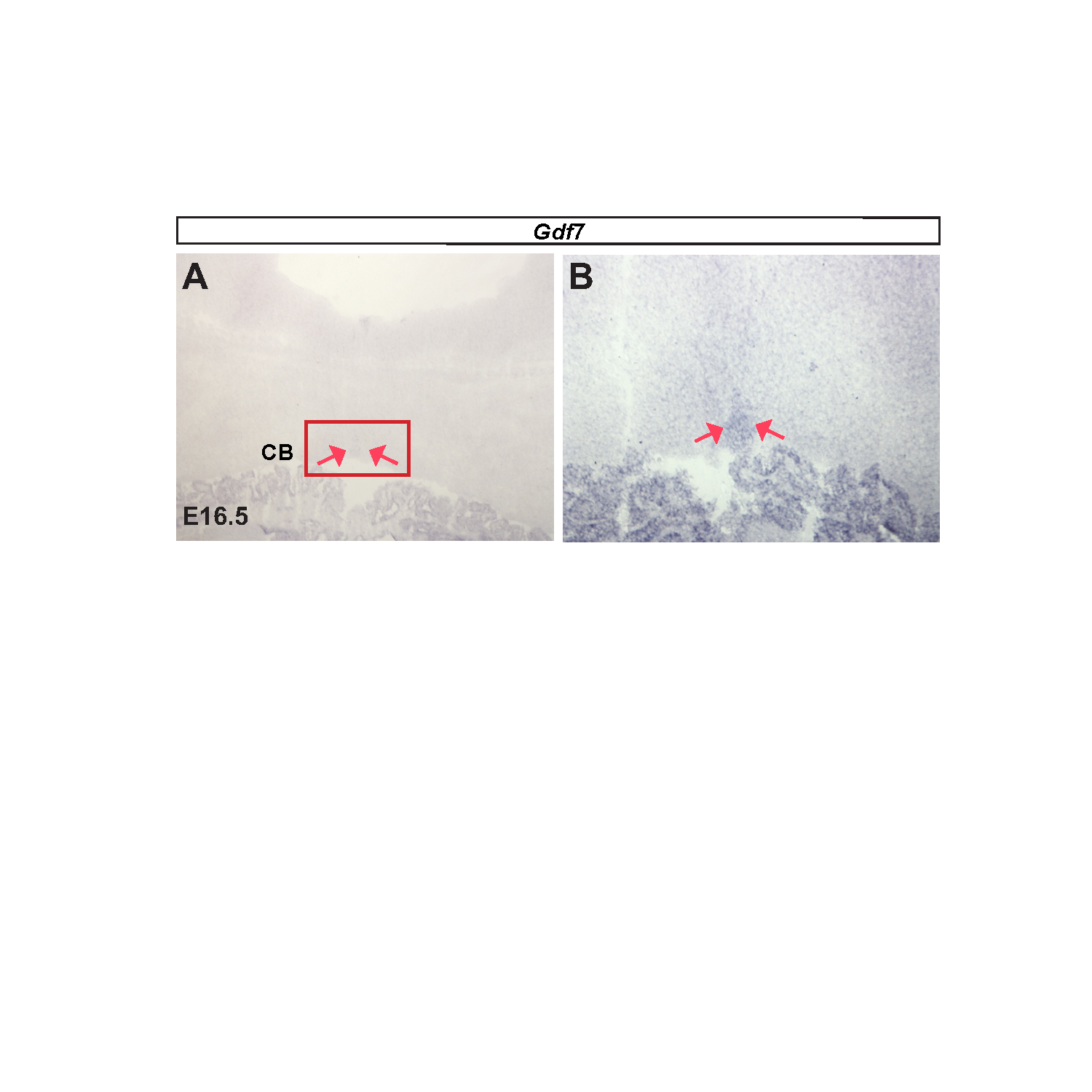

Supplement: Figure S1 — (A–B) In situ hybridization at E16.5 shows Gdf7 expression persists in the cerebellar vermis. (TIFF) [file pone.0035541.s001.tiff]
